# A moving liver phantom in an anthropomorphic thorax for SPECT MP imaging

**DOI:** 10.1007/s13246-021-01081-4

**Published:** 2022-01-01

**Authors:** S. Panagi, Α. Hadjiconstanti, G. Charitou, D. Kaolis, I. Petrou, C. Kyriacou, Y. Parpottas

**Affiliations:** 1 Frederick Research Center, Nicosia, Cyprus; 2 Department of Mechanical Engineering, Frederick University, Nicosia, Cyprus; 3 Department of Medical Physics, Nicosia General Hospital, State Health Services Organisation, Nicosia, Cyprus; 4 Department of Nuclear Medicine, Nicosia General Hospital, State Health Services Organisation, Nicosia, Cyprus; 5 Department of Electrical & Computer Engineering & Informatics, Frederick University, Nicosia, Cyprus

**Keywords:** Liver phantom, SPECT/CT MPI, Cranio-caudal respiratory motion, MP artifacts, Liver activity, Supine and prone imaging

## Abstract

Cranio-caudal respiratory motion and liver activity cause a variety of complex myocardial perfusion (MP) artifacts, especially in the inferior myocardial wall, that may also mask cardiac defects. To assess and characterise such artifacts, an anthropomorphic thorax with moving thoracic phantoms can be utilised in SPECT MP imaging. In this study, a liver phantom was developed and anatomically added into an anthropomorphic phantom that also encloses an ECG beating cardiac phantom and breathing lungs’ phantom. A cranio-caudal respiratory motion was also developed for the liver phantom and it was synchronised with the corresponding ones of the other thoracic phantoms. This continuous motion was further divided into isochronous dynamic respiratory phases, from end-exhalation to end-inspiration, to perform SPECT acquisitions in different respiratory phases. The new motions’ parameters and settings were measured by mechanical means and also validated in a clinical environment by acquiring CT images and by using two imaging software packages. To demonstrate the new imaging capabilities of the phantom assembly, SPECT/CT MP acquisitions were performed and compared to previous phantom and patients studies. All thoracic phantoms can precisely perform physiological motions within the anthropomorphic thorax. The new capabilities of the phantom assembly allow to perform SPECT/CT MP acquisitions for different cardiac-liver activity ratios and cardiac-liver proximities in supine and, for first time, in prone position. Thus, MP artifacts can be characterised and motion correction can be performed due to these new capabilities. The impact of artifacts and motion correction on defect detection can be also investigated.

## Introduction

Myocardial perfusion imaging (MPI) is subjected to a variety of artifacts that can limit the performance of the study. Artifacts can arise at any stage in the MPI process, and they can be grouped into issues related to the patient, equipment, or technologist [1].

Patient-related artifacts in MPI, not originated from the heart, are mainly due to attenuation, thoracic motions, and sub-diaphragmatic activity. Attenuation artifacts are caused due to the anatomy surrounding the heart [2]. Motion artifacts cause blurring along the motion direction, and they are mainly due to the cranio-caudal respiratory motion of the heart [3]. Sub-diaphragmatic organs adjacent to the heart, mainly the liver, present prominent radioactivity which mainly interferes with the adjacent inferior wall of the left ventricle due to photon scattering [4]. The radioactivity of the moving heart and liver, during the cranio-caudal respiratory motion, overlap and as a result it artificially decreases or increases the uptake in the adjacent regions of the heart [5]. Different amplitudes of this motion may result in different spillover of the liver activity into the adjacent heart walls. The liver activity, proximity to the heart and motion amplitude during respiration are the main contributing factors to MP artifacts due to liver [5]. This varying liver interaction causes a complex and clinically unpredictable variation in MPI which may mask cardiac defects in the case of an uptake increase or cause artifactual defects in the case of an uptake decrease or may create a complex combination of these two cases.

Phantoms that closely simulate tissues, anatomy and motion are important to investigate these artifacts and optimise the MP imaging technique, without exposing patients to radiation [6, 7]. To investigate this varying liver interaction and its impact on defect detection, phantoms should be able to simulate various (a) cardiac-liver activity ratios (CLA), (b) cardiac-liver proximities (CLP) and (c) oscillatory amplitudes during respiration.

In single-photon emission computed tomography (SPECT) MPI, the ^99m^Tc extra-cardiac activity (ECA) due to liver was studied using static and manually moving torso phantoms with static cardiac and liver compartments [4, 5, 8, 9]. Germano et al. [4] investigated ECA using a static torso phantom with an enclosed custom-made lucite wedged-shaped liver compartment for three different values of CLA. Nuyts et al. [8], using a static perspex cylinder with an enclosed plastic water-bag liver compartment, studied MP artifacts due to ECA for a CLA value. Heller et al. [9], using a similar phantom to Nuyts et al. [8], also studied MP artifacts due to ECA for a higher CLA value. Pitman et al. [5], using an acrylic cylinder with an enclosed static saline-bag as a liver compartment, examined the effects of ECA interaction for two different CLA values and two different diaphragmatic motion amplitudes by manually moving the cylinder during SPECT MP acquisitions.

In our previous studies [2, 3, 10–12], we acquired SPECT/CT images using an anthropomorphic thorax which enclosed moving thoracic compartments: (a) a cardiac phantom of an ECG beating and moving left ventricle during respiration in the cranio-caudal direction and (b) a breathing phantom of a pair of lungs. These motions could be controlled in time interval of 0.1 s and many parameters of the motions could be varied such as the ejection fraction, heart rate, breath rate and tidal volume. In these studies, we characterised MP artifacts, evaluated physicians reports on cardiac defects and corrected the MP images for the respiratory motion.

The purpose of this paper is to present the development of a liver phantom with motion, implemented within an anthropomorphic thorax and synchronised with the existing thoracic motions, the validation of the new motions’ parameters and settings of the phantom assembly in a clinical environment, and to demonstrate the new imaging capabilities of the phantom assembly in MP SPECT/CT imaging.

## Materials and methods

### Liver phantom

A liver phantom of 1140 mL was designed taking into consideration the liver density, shape and size as well as its position within the thorax and proximity to the heart. For this purpose, the MeshMixer [13] and 3D Slicer [14] design software packages were used. To be able to simulate various cardiac-liver activity ratios during SPECT/CT acquisitions, the liver phantom was designed as a hollow cavity with a wall thickness of 2 mm. Two holes were added to the design to fill the phantom with water and radiopharmaceutical, with diameters of 20 and 4 mm, respectively. A diagonal base was also added to the design, at the outer inferior border of the liver, to be able to glue a thread and then tight the rod for holding the liver in position within the thorax.

**Fig. 1:**
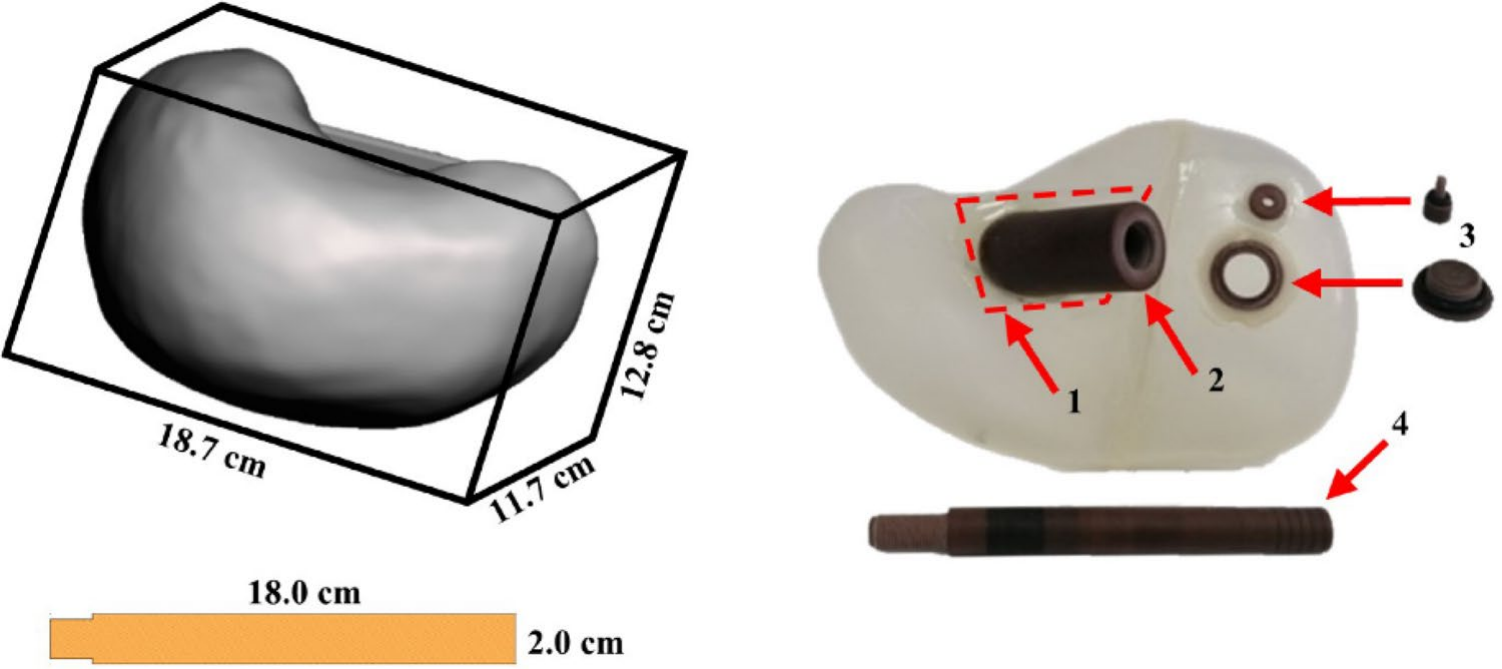
(Left) The 3D design of the liver phantom together with its dimensions, and (right) the manufactured liver phantom with the (1) diagonal base (red dashed shape), (2) thread, (3) caps and (4) rod. Two opposite views (left and right) of the phantom are shown

The liver phantom was manufactured by using the Form2 3D-printer (FormLabs) and a transparent resin (Clear Resin V4) material with a density of 1.06 g/cm^3^. The sealing caps for the holes, the thread and the rod were manufactured from a solid-water material. Figure 1 (left) shows one side of the designed liver together with its dimensions, as given by the printing software package, while Fig. 1 (right) shows the other side of the manufactured liver phantom.

### Motion mechanism

**Fig. 2:**
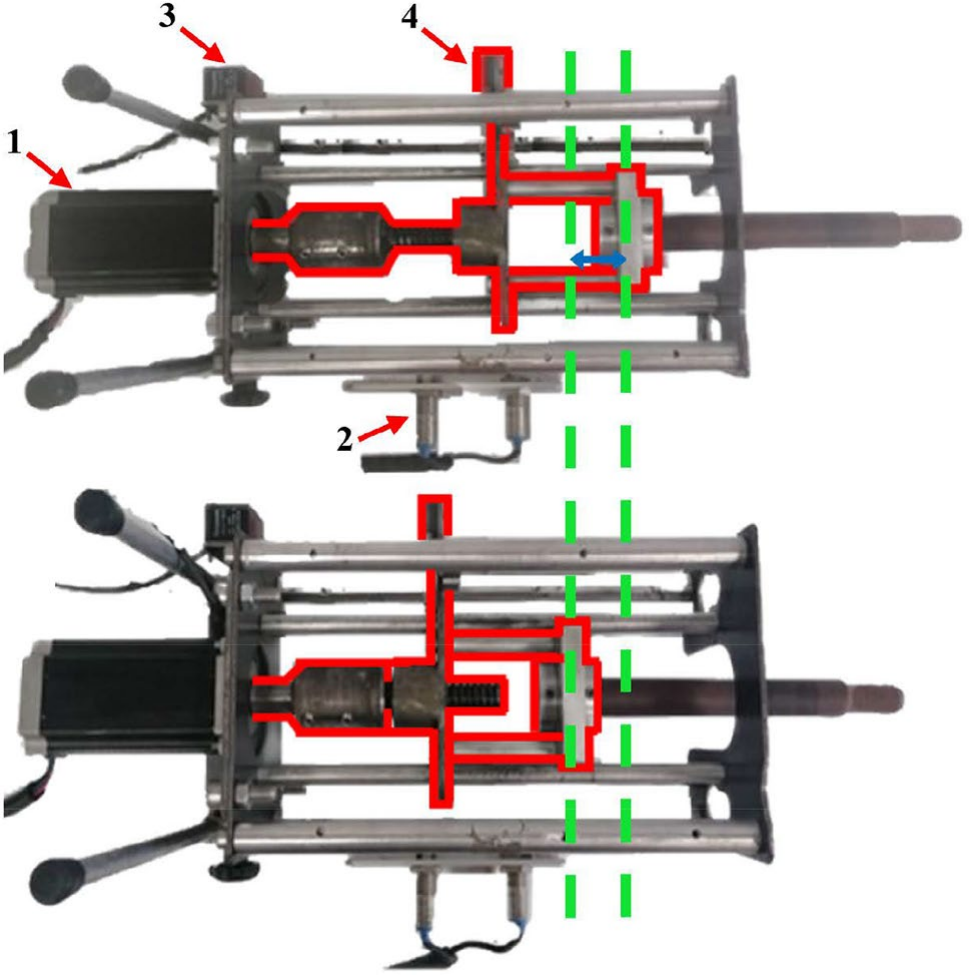
Top view of the mechanism for the cranio-caudal liver motion. The threaded-end of the rod is shown at the two extreme amplitudes of the motion where the liver phantom is at its (top) uppermost and (bottom) lower positions within the thorax. The main components of the motion are also shown: (1) the motor, (2) the proximity sensors, (3) the laser sensor and (4) its reflector. The screwed-rod system is outlined with a red colour. The green dotted lines and the blue double-arrow line indicate the amplitude of the motion

**Fig. 3:**
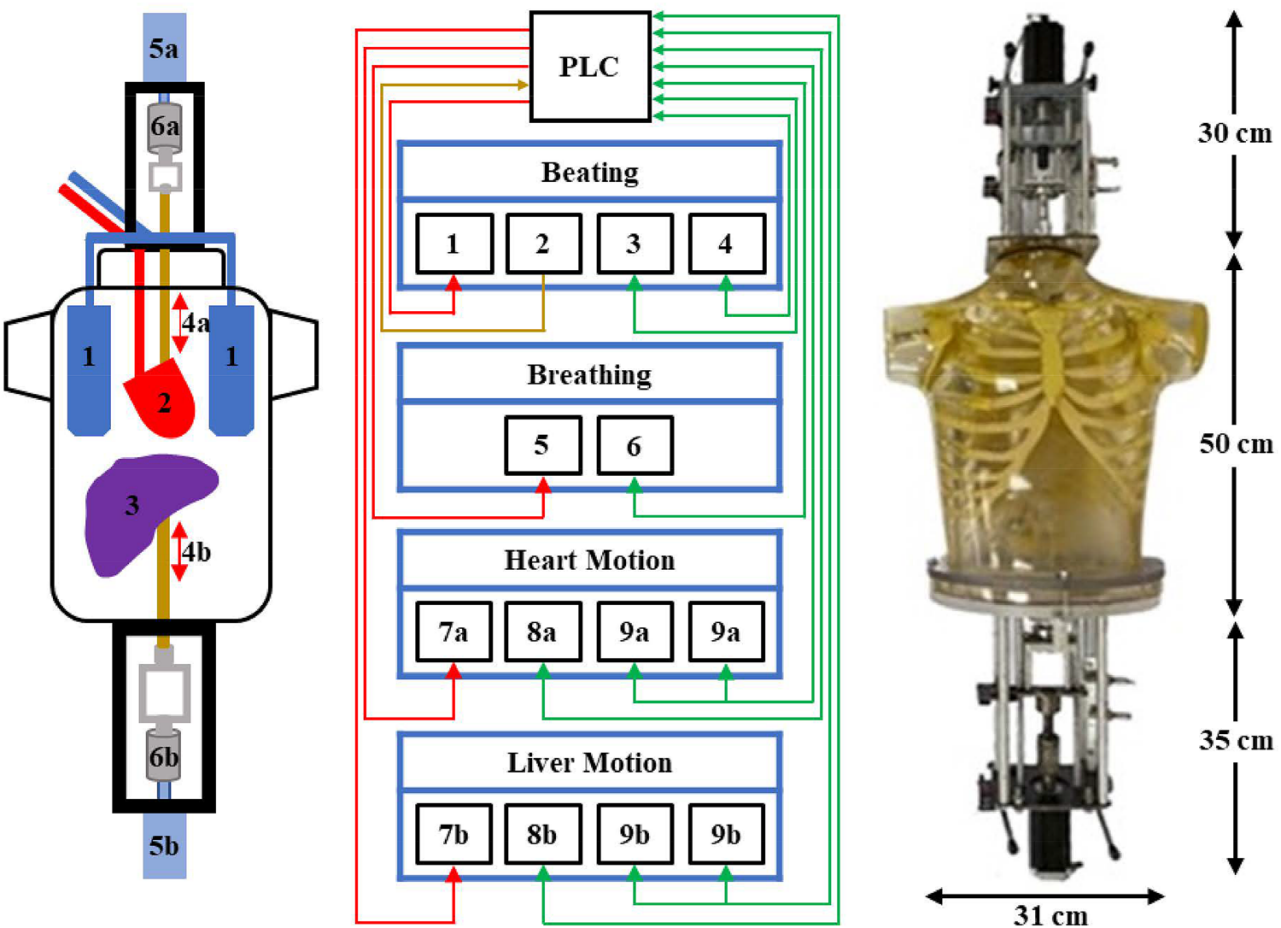
(Left) A schematic diagram of the anthropomorphic thorax with the enclosed (1) lungs, (2) cardiac, (3) liver phantoms, and the attached mechanisms to support the cranio-caudal (4-6a) cardiac and (4-6b) liver motions during respiration: (4) cardiac and liver rods, (5) motors and (6) screwed-rod systems (grey colour). (Middle) A basic circuit among the PLC and the devices for beating, breathing, cardiac and liver motions: (1, 7) drivers of the corresponding motors, (2) ECG simulator, (3) rotary sensor, (4) position sensor, (5) proportional valve, (6) pressure sensor, (8) laser distance sensors and (9) proximity sensors. The PLC sends signals to the devices (red colour wires) or receives a signal from the device (brown wire) while the sensors provide feedback to the PLC to control the motions (green wires). (Right) The dimensions of the anthropomorphic thorax and of the attached mechanisms

A mechanism, controlled by a touch-screen programmable logic controller (PLC), was manufactured to provide motion to the liver phantom. The motion was transferred via the rod which at one end, in the thorax, was attached to the phantom (Fig. 1) and at the other end, outside the thorax, was attached to the mechanism (Fig. 2). The rod entered the thorax via a water-tight hole at the base of the thorax. The mechanism and the rod were aligned with that hole, and then the mechanism was attached at the outer base of the thorax phantom (Fig. 3).

Figure 2 shows the main components of the mechanism to generate the liver motion: (a) the motor, (b) the screwed-rod system, (c) the laser distance sensor with its reflector and (d) the two proximity sensors. The PLC drove the motor to oscillate outwards-inwards the screwed-rod system and the rod of the liver phantom, as seen in the figure. The laser sensor was directly connected to the PLC providing feedback about the motion. Note that the laser distance sensor was stable while its reflector was moving together with the screwed-rod system. Two proximity sensors were positioned on the mechanism, vertical to the motion of the screwed-rod system and 3-cm apart from each other, to define the minimum and maximum limits of the motion amplitude. These sensors serve as safe interlocks for the motion.

The cardiac-liver proximity was defined by the length of the liver rod that entered in the thorax. This proximity could be set between 5 and 25 mm in distance intervals of 5 mm. For this purpose, the rod could be properly tightened on the mechanism using the corresponding drawn marks on the rod. For prone imaging, this motion mechanism together with the rest of the phantom assembly could be turned by 180°.

A schematic diagram of the anthropomorphic thorax with the enclosed thoracic phantoms and the attached mechanisms for the cranio-caudal cardiac and liver motions during respiration is shown in Fig. 3 (left). The mechanisms for beating and breathing were previously presented and described [12]. The mechanism for the cranio-caudal cardiac motion during respiration is similar to the corresponding one of the liver phantom. The dimensions of the thorax and of the attached mechanisms are shown in Fig. 3 (right). A basic circuit among the PLC and the devices for the four main motions of the phantom assembly is shown in Fig. 3 (middle). The PLC sends signals to the devices or receives signals from the devices while the sensors provide feedback to the PLC to control the motions.

### Liver motion

A VisiLogic ladder program was developed in the PLC to describe the cranio-caudal motion of the liver during respiration, and consequently, to control the motion of the motor.

The equation of motion is as follows:

1 $$ {\text{X}}_{{\text{L}}} \left( {\text{t}} \right) = {\text{X}}_{0} \,{\text{sin}}\left( {\alpha {\text{t}} + {9}0^\circ } \right) $$

where *X*_*L*_*(t)* is the displacement (in mm) of the phantom with respect to time, *X*_*o*_ is the maximum amplitude of the oscillation, *t* is the counting time in intervals of 0.1 s, *α* = *2π/Τ*, and *T* is the time-period of the motion.

Note that the same equation was utilised for the cranio-caudal cardiac motion during respiration [12]. The counting time and time-period of the liver and cardiac motions were synchronised with the breathing equation. At *t* = *0 s*, the lungs’ phantom is at end-exhalation and the cardiac-liver phantoms are in the corresponding anatomical positions, at their uppermost positions in the thorax (0 mm). At half of the time-period defined by the breath rate, the cardiac-liver phantoms following Eq. 1 are at their lowest positions in the thorax while the lungs following the breathing equation [12] are at end-inspiration. Note that the inferior portion of the lungs’ phantom at the level of the diaphragm follows the cardiac and liver phantoms during their cranio-caudal oscillatory motion. For a defined breath rate, all phantoms can perform normal or deep respiratory motion. The maximum displacement of the cardiac-liver phantoms from their uppermost position in the thorax is 15 or 27 mm for normal or deep breathing, respectively, while the tidal volume in the lungs is 450 or 550 mL for normal or deep breathing, respectively.

In addition to the abovementioned synchronised PLC mode of continuous motion, a synchronised PLC mode of dynamic respiratory phases was also developed. The respiratory motion was previously divided into four static phases from end-exhalation to end-inhalation [2, 3, 12]. The displacement of the cardiac-liver phantoms from their uppermost position in the thorax and the volume of the lungs at each static phase, for normal and deep breathing, are presented in Table 1. A dynamic respiratory phase was defined between two successive static phases and its motion was performed following the corresponding equations of the phantoms. Three such motions were developed between the four static phases for normal and deep breathing, as seen in Table 1.

**Table 1:** The displacement of the cardiac-liver phantoms from their uppermost position in the thorax and the volume of the lungs when performing synchronised oscillations in the PLC mode of dynamic respiratory phases

Dynamic respiratory phase	Normal breathing			Deep breathing	
	Oscillation between static phases	Lungs’ volume (mL)	Cardiac-liver displacement (mm)	Lungs’ volume (mL)	Cardiac-liver displacement (mm)
1	1 ↔ 2	1100 ↔ 1145	0 ↔ 5	1100 ↔ 1155	0 ↔ 5
2	2 ↔ 3	1145 ↔ 1344	5 ↔ 10	1155 ↔ 1398	5 ↔ 20
3	3 ↔ 4	1344 ↔ 1550	10 ↔ 15	1398 ↔ 1650	20 ↔ 27

### Mechanical measurements

Mechanical means were utilised to measure the accuracy of these new motions added on the phantom assembly. The displacement of the cardiac and liver phantoms during the cranio-caudal continuous or in phases motions was controlled by the internal counter of the PLC (in time intervals of 0.1 s) and the laser distance sensors (in distance intervals of 1 mm). The screen monitor of the laser distance sensors provided feedback by recording this displacement at any time. A timer and a digital sliding caliper were also used to measure and verify the calculated displacement at any time. The displacement error was found to be within the accuracy of the laser distance sensor.

The tidal volume in the lungs at any time during the mode of dynamic respiratory phases was measured with a dedicated custom-made structure [11]. The lungs were immersed and held within a fully filled water tank. The lungs and the breathing mechanism were connected via tubes that could pass through the air-tight openings of the tank. The tidal volume in the lungs could raise or lower the level of water in the graduated cylinder which was attached on the top part of the tank. For normal and deep breathing, and breath rates of 10–14, the tidal volume with respect to time for any of these three motions between the static respiratory phases was measured. It was found that the calculated volume at any time was in agreement with the measured one within the accuracy of the graduated cylinder (± 5 mL).

### CT and SPECT/CT acquisitions

The phantom assembly with the enclosed thoracic phantoms, including the new insert of the liver phantom, and the new motions’ parameters and settings, were validated in a clinical environment by acquiring CT images and using two commercial imaging software packages. The Toshiba Aquilion RXL 16-CT system of the Limassol General Hospital was utilised to perform CT acquisitions of the phantom assembly. The slice thickness was 0.5 mm (1200 slices per image), the tube voltage was set at 120 kV_p_ and the current at 100 mA. The OsiriX [15] and Vitrea [16] software packages were used to measure (a) the liver volume, (b) the different cardiac-liver proximities and (c) the cardiac-liver displacement and the tidal volume in the lungs at the endpoints of each dynamic respiratory phase.

SPECT/CT acquisitions were also performed to demonstrate the new capabilities of the phantom assembly in MPI. A nuclear medicine physician reported on the images without having any prior knowledge of the motions’ parameters and settings of the phantom assembly and of the acquisition and processing protocols. These reports were compared with previous phantom and patients studies.

The GE Discovery-670-DR SPECT/16-slice-CT of the Nicosia General Hospital was utilised to perform the acquisitions of the phantom assembly, in supine and prone position, for different motions’ parameters and settings. The acquisitions were performed using a routine clinical protocol with two Low-Energy High-Resolution collimators in 90° (L-mode) orientation. Data were acquired in 60 projections, 20 s per projection, over 180° of rotation, with a matrix of 64 × 64. An activity of 15 MBq of ^99m^Tc was injected into the myocardium wall of the left ventricle, based on an estimate of 1.2% uptake of a clinically relevant administered activity [17]. Also, an activity of 7.5 MBq of ^99m^Tc was injected within the liver phantom to achieve a cardiac-liver activity ratio of 1:0.5 which is common clinical scenario. A 20% energy window was centred over the 140 keV photopeak of ^99m^Tc. CT images were acquired around the heart region, with 5 mm slice thickness and a matrix of 512 × 512. SPECT data were reconstructed using the ordered subset expectation maximisation algorithm with 2 iterations and 10 subsets. A Butterworth filter (cut-off: 0.52, power: 5) was applied on the reconstructed images. SPECT data were scattered and attenuated corrected.

## Results

**Fig. 4:**
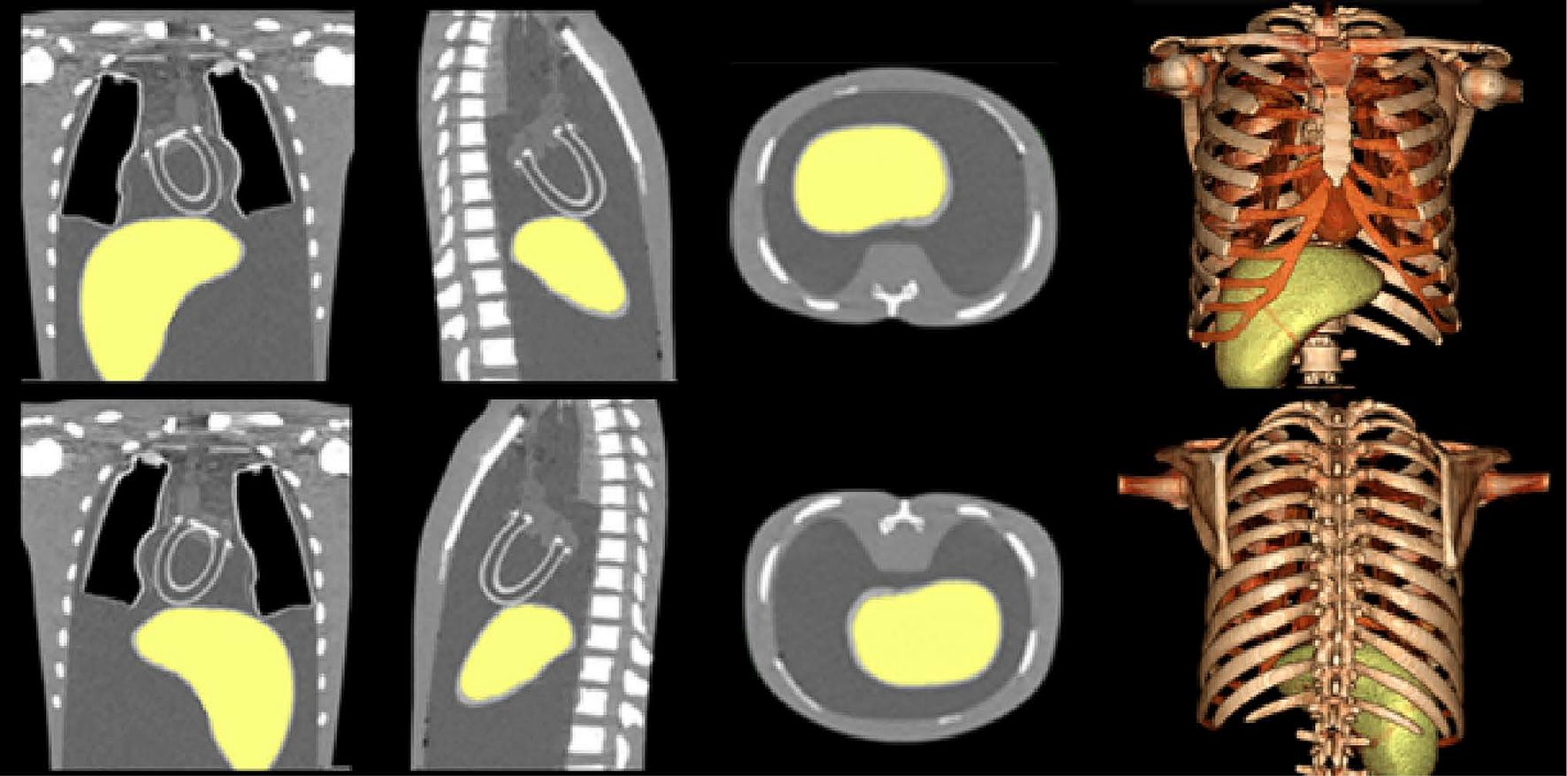
Coronal (1st column), sagittal (2nd column), axial (3rd column) slices and 3D views (4th column) of the phantom assembly in supine (top row) and prone (bottom row) positions. The images were acquired by the Toshiba Aquilion RXL 16-CT modality and processed by using the Vitrea software

Figure 4 shows coronal, sagittal and axial CT slices as well as 3D views of the thorax with its enclosed thoracic phantoms, in supine and prone positions. The CT images were processed by using the Vitrea software. The liver phantom within the thorax is presented with a yellow colour. The liver volume was measured with the Vitrea software to be 1150 ± 25 mL.

**Fig. 5:**
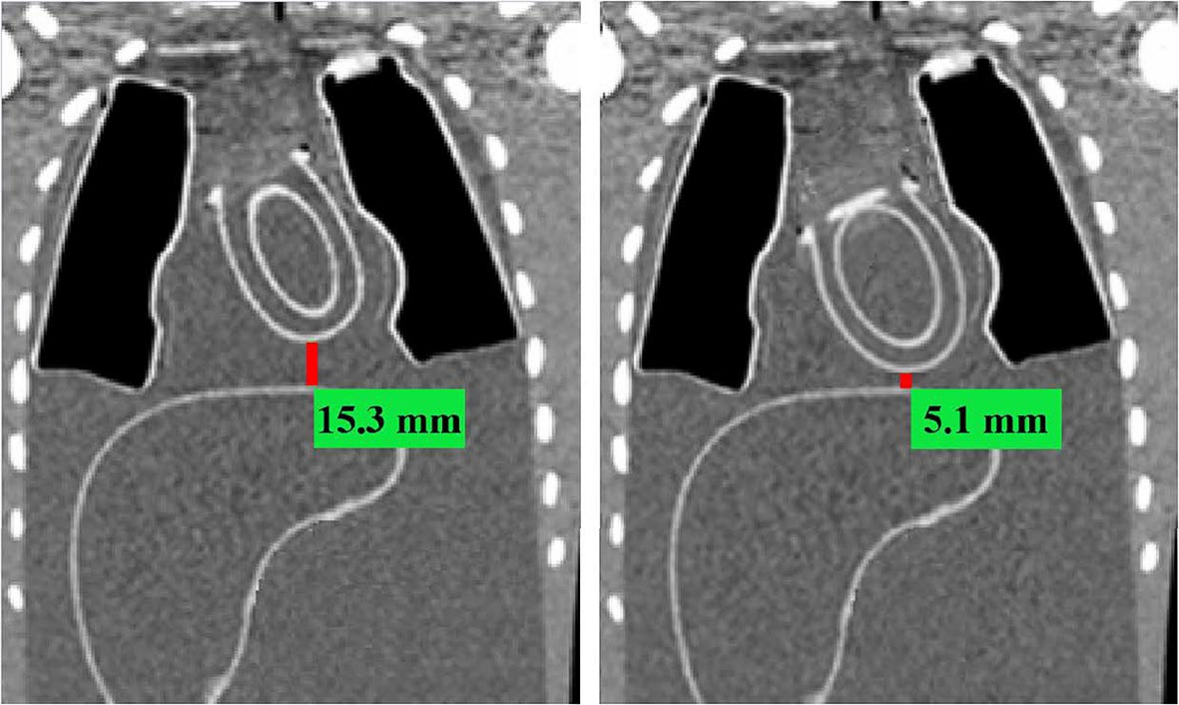
The cardiac-liver proximities when the cardiac phantom was at systole (left) and at diastole (right). The cardiac-liver proximity was mechanically set to be 5 ± 1 mm when the phantom was at diastole. The measured values from the OsiriX software are shown in the figure

Figure 5 shows coronal CT slices of the phantom assembly when the cardiac phantom was at systole and at diastole. The cardiac-liver proximity was mechanically set to be 5 ± 1 mm when the cardiac phantom was at diastole, and it was measured with the OsiriX software to be 5.1 ± 1 mm. The corresponding distance when the cardiac phantom was at systole was measured to be 15.3 ± 2 mm. Note that the beating cardiac phantom contracts by about 10 mm. All possible abovementioned mechanically set cardiac-liver proximities were also measured with the OsiriX software when the cardiac phantom was at diastole and at systole. The maximum deviation from the marked values on the rod was 3 mm which was within the measured errors.

**Fig. 6:**
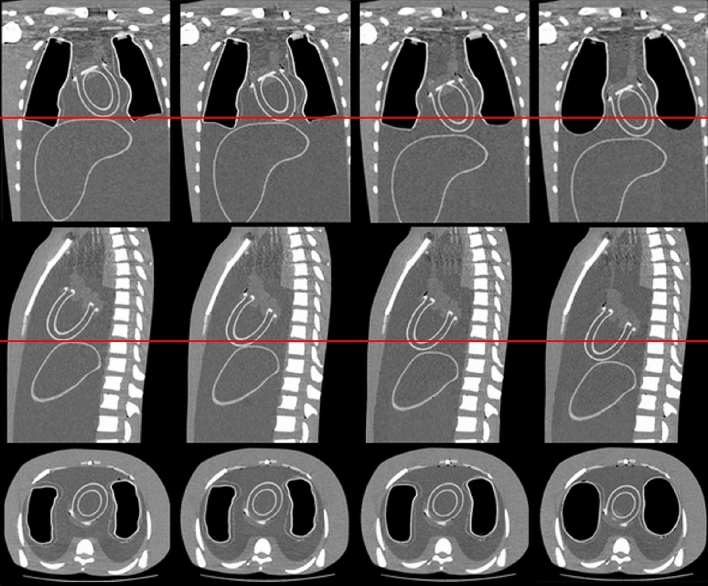
Coronal (top row), sagittal (middle row) and axial (bottom row) slices of the phantom assembly at the endpoints of the three isochronous dynamic respiratory phases (four static phases) from end-exhalation to end-inhalation, in deep breathing: phase-1 (1st column), phase-2 (2nd column), phase-3 (3rd column) and phase-4 (4th column). The downward displacement of the thoracic phantoms is indicated with the red lines

Figure 6 shows the coronal (top row), sagittal (middle row) and axial (bottom row) slices of the phantom assembly at the endpoints of the three isochronous dynamic respiratory phases (four static phases) from end-exhalation to end-inhalation, in deep breathing. The downward displacement of the thoracic phantoms is indicated with the red lines on the coronal and sagittal slices. The CT images at the endpoints of the dynamic respiratory phases, in normal and deep breathing, were utilised to measure with the OsiriX software package the volume of the lungs and the downward displacement of the thoracic phantoms. A 2% maximum deviation was calculated between the measured volumes and the calculated values of Table 1, as in refs [11, 12]. For the downward displacements, a 1.7% maximum deviation was calculated between the measured and the calculated values of Table 1.

**Fig. 7:**
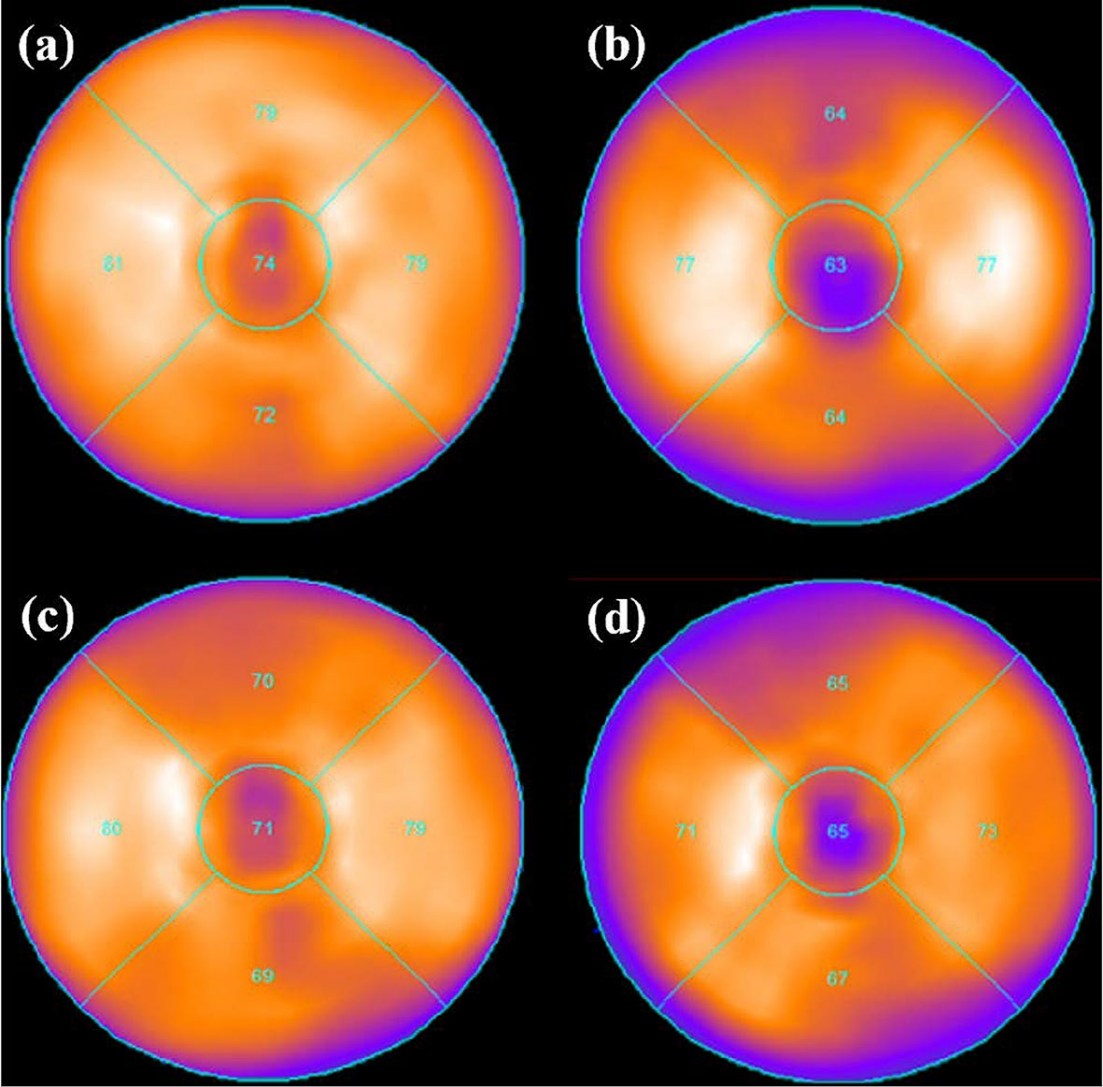
5-segments polar maps from the SPECT/CT acquisitions of the phantom assembly for the following settings: an ECG beating left ventricle, without cardiac defects, a CLA of 1:0.5, in supine position and CLP of 5 mm (**a** and **b**), in prone position and CLP of 20 mm (**c** and **d**), without cranio-caudal motion (**a** and **c**), in deep (**b**) and in normal cranio-caudal respiratory motion (**d**)

Figure 7 shows 5-segments polar maps from the SPECT/CT acquisitions of the phantom assembly with the following settings: an ECG beating left ventricle, without cardiac defects, a CLA of 1:0.5, in supine position and CLP of 5 mm (a and b), in prone position and CLP of 20 mm (c and d), without cranio-caudal motion (a and c), in deep (b) and in normal cranio-caudal respiratory motion (d). Note that the CLP values are given for the cardiac phantom at diastole. The prone imaging was performed with a CLP of 20 mm while the supine imaging with a CLP of 5 mm since in prone position the inferior myocardial wall and the diaphragm are well separated [18]. Also, the prone imaging was performed for normal while the supine imaging for deep respiratory motion since prone position reduces the degree of patient motion [19].

A nuclear medicine physician visually evaluated the images and reported an uptake decrease in the anterior and inferior regions of the myocardial wall of the left ventricle, in both the supine and prone imaging, when the thoracic phantoms were performing cranio-caudal respiration motion. However, the uptake decrease in the inferior region was higher when the phantom assembly was in the supine than in the prone position.

## Discussion

A liver phantom was designed, manufactured and anatomically added in an anthropomorphic thorax. A continuous liver motion was developed and synchronised with the cranio-caudal respiratory motion of the other existing thoracic phantoms. In addition, three synchronised motions between the static respiratory phases, from end-exhalation to end-inhalation, were developed for all the thoracic phantoms. These motions are precisely controlled by the PLC equations, its internal counter and the laser distance sensors. Mechanical means were also utilised to measure the cranio-caudal displacement of the thoracic phantoms and the tidal volume in the lungs with respect to time.

The phantom assembly with all the enclosed thoracic phantoms and the new motions’ parameters and settings, were validated in a clinical environment by acquiring CT images and using two commercial imaging software packages. First, the anatomical position of the liver phantom within the thorax was verified in supine and prone positions by using the Vitrea software. Second, the OsiriX software was used to measure (a) the liver volume, (b) the cranio-caudal displacements of the thoracic phantoms and the tidal volumes in the lungs at the endpoints of each dynamic respiratory phase, for different breath rates in normal and deep breathing, and (c) all possible mechanically set cardiac-liver proximities when the cardiac phantom was at diastole and at systole. The measured values were compared with the calculated ones from the PLC equations and a small deviation was reported.

SPECT/CT acquisitions were also performed to demonstrate the new capabilities of the phantom assembly in MPI. In particular, a specific activity of ^99m^Tc was injected into the liver phantom to achieve a cardiac-liver activity ratio of 1:0.5, and the phantom assembly was imaged for two different cardiac-liver proximities in supine and prone positions. A nuclear medicine physician reported that, in both the supine and prone imaging, motion artifacts were induced in the anterior and inferior myocardial walls. However, in prone imaging, the inferior artifact was reduced due to less diaphragmatic attenuation [18, 19]. These are in agreement with previous phantom and patients studies [2, 18, 19].

The previous motions and imaging capabilities of the phantom assembly were described in refs [2, 3, 10–12]. It was utilised to characterise various types of SPECT/CT MP artifacts in supine imaging and investigate their impact on defect detection [2]. The static respiratory phases of the phantom assembly were used to perform SPECT/CT acquisitions in supine position, apply a motion correction algorithm to correct the images for the cranio-caudal cardiac motion during respiration, and investigate the impact of motion correction on defect detection [3].

The liver phantom, and the new motions’ parameters and settings, added new imaging capabilities to the phantom assembly so as to perform SPECT/CT acquisitions for different cardiac-liver activities ratios and for different cardiac-liver proximities in prone and supine positions. These capabilities of the phantom assembly allow to characterise more types of MP artifacts and investigate their impact of defect detection. Acquisitions of the phantom assembly can also be performed in each of the dynamic respiratory phases rather than in each of the static respiratory phases [3] allowing to correct the images for the cranio-caudal cardiac motion during respiration by applying a more realistic scenario.

Only another one SPECT MPI study [5] was performed with a moving liver phantom. In that study, an acrylic cylinder, which enclosed a static and non-beating cardiac phantom and a static saline-bag as a liver compartment, was manually moving during acquisitions. A SPECT/^153^Gd modality was used and that phantom was imaged in supine position. A more realistic scenario is presented with this anthropomorphic phantom where the ECG beating cardiac, the breathing lungs and the liver phantoms precisely move within the thorax during respiration rather than manually moving a cylinder with static compartments. In addition, for the first time, an anthropomorphic thorax with moving thoracic phantoms can be imaged not only in supine but also in prone position.

Even though a number of motion limitations of other phantoms have been overcome and the main motions are precisely performed, this phantom assembly does not simulate the small heart shifts in other directions during respiration, the heart twisting and the chest wall expansion.

## Conclusions

We have presented the development of a liver phantom which was implemented within an anthropomorphic thorax together with the existing thoracic phantoms of the ECG beating left ventricle and the inflatable lungs. All thoracic phantoms were synchronised to perform the cranio-caudal respiratory motion. This oscillatory motion can be continuous between predefined amplitudes for the cardiac-liver phantoms and tidal volumes for the lungs, in normal or deep breathing. This motion can also be performed between two successive static respiratory phases, from end-exhalation to end-inhalation, with predefined amplitudes and tidal volumes, in normal or deep breathing. All motions are controlled by a PLC in time interval of 0.1 s and by precise sensors. Several motion limitations of other phantoms have been overcome.

The new motions’ parameters and settings of the phantom assembly were validated in a clinical environment using CT images and commercial imaging software packages. The new imaging capabilities of the phantom assembly in SPECT/CT MP imaging were also demonstrated. It can be utilised to perform SPECT MP acquisitions for different cardiac-liver activity ratios and cardiac-liver proximities in supine and prone positions.

Future studies with this phantom assembly will include characterisation of MP artifacts and correction of the images for the cranio-caudal cardiac motion during respiration. The impact of artifacts and of motion correction on cardiac defect detection will also be investigated.
